# Use of polyhexamethylene biguanide on wound healing in dogs

**DOI:** 10.1590/acb413026

**Published:** 2026-06-29

**Authors:** João Vitor dos Santos Alves da Silva, Brenda Mendonça de Alcântara, Natália Helena Meneguin, Sergio Andrés Millán Guaita, Tryssia Scalon Magalhães Moi, Gabriel João Unger Carra, Josiane Morais Pazzini, Bruno Watanabe Minto

**Affiliations:** 1 Universidade Estadual Paulista – School of Agrarian and Veterinary Sciences – Department of Pathology, Reproduction and One Health – Jaboticabal (SP) – Brazil.; 2 Universidade Estadual Paulista – School of Agrarian and Veterinary Sciences – Department of Veterinary Clinic and Surgery – Jaboticabal (SP) – Brazil.; 3 Universidade de São Paulo – School of Veterinary Medicine and Animal Science – Department of Surgery – São Paulo (SP) – Brazil.; 4 Universidade Brasil – Veterinary Medicine Program – Department of Veterinary Surgery – Fernandópolis (SP) – Brazil.

**Keywords:** Biguanides, Dogs, Surgical Wound

## Abstract

**Purpose:**

To analyze the effects of polyhexamethylene biguanide (PHMB) on healing of clean surgical wounds in dogs.

**Methods:**

Thirty healthy, non-neutered dogs of different breeds underwent elective ovariohysterectomy or orchiectomy, and a 1-cm circular abdominal wound was created. Animals were divided into three groups (n = 10). Groups T1 and T2 received PHMB dressings changed once and twice daily. The control group (C) had wounds cleaned daily with saline and covered with gauze. Macroscopic evaluations were performed daily for 10 days, assessing edema, exudate, cosmetic appearance, and color. After 10 days, excisional biopsies were collected for histopathology evaluating vascular proliferation, inflammatory cells, fibroblastic proliferation, collagenization, necrosis, hyperplasia, and reepithelialization, and immunohistochemistry for reepithelialization and angiogenesis.

**Results:**

Macroscopic evaluations showed no significant differences among groups. Histopathology revealed greater collagenization in group C than in T2 (*p* < 0.05), while T1 did not differ from the others. Immunohistochemistry showed no differences in reepithelialization among groups. However, angiogenesis was higher in C and T2 than in T1 (*p* < 0.05).

**Conclusion:**

PHMB dressings neither demonstrate superior healing compared with gauze and saline cleaning nor impair healing and may represent an option for wound management in dogs.

## Introduction

The wound healing process in dogs is highly complex and is primarily divided into the phases of inflammation, proliferation, and remodeling, involving diverse signaling pathways and cellular activities^1,2^.

In veterinary medicine, a wide variety of techniques and products aimed at improving the wound healing process are described, each with multiple advantages and disadvantages. Topical therapies are widely available, ranging from ointments containing active ingredients with healing, antiseptic, and/or anti-inflammatory potential to formulations with growth factors, acellular dermal matrices, allografts, and cell-based therapies^3^. However, no product or dressing is considered the gold standard in veterinary medicine, underscoring the need and relevance of further studies exploring alternative products or techniques.

An interesting alternative in wound treatment is polyhexamethylene biguanide (PHMB), or polyhexanide, a polymer used as a broad-spectrum antimicrobial agent^4^. Its applications are highly diverse, showing promising results in general industry and gaining significant attention for its medical applications^5-7^.

Studies in different animal models, such as rats, rabbits, dogs, and piglets, as well as *in-vitro* and human trials, have shown that PHMB is a broad-spectrum antiseptic with high tissue compatibility and low toxicity. It is available in various forms, including ointments, gels, foams, and dressings. Its antimicrobial effect results from disrupting the bacterial membrane and inhibiting cellular metabolism through strong interactions with negatively charged phospholipids in bacterial membranes^8^.

In dogs, wound lavage with PHMB and saline in bite wounds significantly reduced the bacterial load and ensured both immediate and complete decontamination^9^. Although evidence from human medicine shows that PHMB is effective in improving chronic wound healing, reducing bacterial burden, and alleviating wound-related pain when applied topically, as well as exhibiting activity against fungi, viruses, and protozoa^10,11^, these findings cannot be directly extrapolated to veterinary patients. Therefore, considering the species-specific aspects of wound healing, studies specifically evaluating the effects of PHMB on wound healing in dogs remain limited and are necessary to better support its clinical use in veterinary medicine.

The objective of this study was to evaluate the therapeutic effects of PHMB, emphasizing on wound healing quality, compared to treatment based on cleansing and dry gauze dressing in clean surgical canine wounds.

## Methods

The methodology of this study was approved by the Animal Use Ethics Committee of the Faculty of Agricultural and Veterinary Sciences, Universidade Estadual Paulista “Júlio de Mesquita Filho” (UNESP), Jaboticabal, SP, Brazil (Protocol No. 010177/19). The study was conducted at the “Governador Laudo Natel” Veterinary Hospital located at the same institution.

Thirty dogs (20 females and 10 males) were used in the study, all unneutered, of various breeds, healthy, and not receiving any medication. The animals underwent elective ovariohysterectomy or orchiectomy procedures and simultaneously had a circular skin lesion created in the abdominal region using a 1-cm punch. They were divided into three experimental groups of 10 animals each:

- Group 1 (T1) had an average age of 31.9 months old (ranging from 10 to 120 months old);
- Group 2 (T2) had an average age of 54.7 months old (ranging from 9 to 120 months old);
- Group 3 (C) had an average age of 37.2 months old (ranging from 12 to 72 months old).

Animals in the T1 and T2 groups had their wounds cleaned with 0.9% saline solution and received PHMB-impregnated dressings, changed once or twice daily, respectively. In the C group, wound cleaning was also performed with 0.9% saline solution and covered with dry gauze (control group) once a day.

Macroscopic evaluations of the healing process were conducted once daily until the 10-day mark for all groups. A score (Table 1) was assigned based on the presence of edema, erythema, pain/discomfort, vocalization upon manipulation, pruritus, secretion, amount of secretion, and healing. At the end of the 10-day period, an excisional biopsy was performed on the wound healing area for histopathological evaluation, allowing analysis of microscopic characteristics (vascular proliferation, mononuclear cells, polymorphonuclear cells, fibroblastic proliferation, collagenization, necrosis, hyperplasia, and reepithelialization) (Tables 2 and 3) and immunohistochemistry (reepithelialization and angiogenesis).

**Table 1:** Scoring system for macroscopic evaluation of surgical wounds.

Variables/points	0	1	2	3	4
Presence and degree of edema	Absent	Mild	Moderate	Severe	-
Presence and degree of erythema	Absent	Mild	Moderate	Severe	-
Presence and degree of pain/discomfort	Absent	Mild	Moderate	Severe	-
Vocalization upon manipulation	Absent	Mild	Moderate	Severe	-
Presence and degree of pruritus	Absent	Mild	Moderate	Severe	-
Presence and characterization of secretion	Absent	Serous	Ceruminous	Purulent	Mucoid
Amount of secretion	Absent	Mild	Moderate	Severe	-
Healing progression	-	Good	Fair	Poor	-

Source: adapted from Minto et al.^13^.

**Table 2:** Scoring system for microscopic evaluation of surgical wounds.

Variables/Points	0	1	2	3
Collagenization	Absent	Mild	Moderate	Severe
Angiogenesis	Absent	Mild	Moderate	Severe
Fibroblasts	Absent	Mild	Moderate	Severe
Polymorphonuclear cells	Absent	Mild	Moderate	Severe
Mononuclear cells	Absent	Mild	Moderate	Severe

Source: adapted from Minto et al.^13^.

**Table 3:** Scoring system for histological evaluation of healing process.

Variables/Points	0	1	2	3
Necrosis	Absent	Mild	Moderate	Severe
Hyperplasia	Absent	Mild	Moderate	Severe
Reepithelialization	Absent	Mild	Moderate	Severe

Source: adapted from Minto et al.^13^.

The histopathology samples were fixed in 10% formalin, embedded in paraffin, sectioned at 5 µm, stained with hematoxylin-eosin, and analyzed under a light microscope.

The immunohistochemistry study aimed to analyze the variables reepithelialization and angiogenesis. The sections were mounted on pre-cleaned, degreased glass slides, prepared with organosilane-based adhesive (3-aminopropyltriethoxysilane, Sigma Chemical C.O., United States of America), and subjected to specific antibodies. The detection system followed the manufacturer’s instructions (Table 4).

**Table 4:** Antibodies used in immunohistochemical reactions on dog skin samples.

Antibody	Clone	Company	Dilution	Antigen retrieval	Incubation period (min)	Detection system
CD31	Monoclonal	Dako, JC70A	1:50	Pepsin porcine gastric mucosa—Sigma Life Science, United Kingdom	120	Novolink polymer detection systems—Leica Biosystems, UK
Ae1/Ae3	Polyclonal	ImPath, Cytokeratin cocktail	1:400	Novocastra epitope retrieval solutions pH9 – Leica Biosystems, UK	120	Novolink polymer detection systems— Leica Biosystems, UK

Source: adapted from Minto et al.^13^.

The antibody staining for Ae1/Ae3 was assessed using photomicrographs obtained with light microscopy at 2× magnification, analyzed with NIS-Elements software (Nikon, version 4.30). The images were analyzed using the Image J software, with the Threshold Colour plugin. The angiogenesis index for CD31 was determined using the microvascular count technique according to Maeda et al.^12^. The areas with the highest vascularization were identified at 40x magnification. Vessel counting was done in five pre-selected fields with high vascular density at 400x magnification using optical microscopy. The microvascular count was determined twice by a single evaluator at different times and expressed as the average number of vessels in each case studied.

This was a randomized, double-blind clinical trial (both the evaluator and the tutor were unaware of which substance was applied to each lesion).

The descriptive analysis of the variables was performed considering the median values and interquartile range. The macroscopic variables, due to having repeated measures over time and being ordinal qualitative variables, were analyzed using cumulative logit models adjusted by Gauss-Hermite with seven quadrature points (cumulative link mixed model), an extension of the generalized linear mixed models. The set of microscopic variables was analyzed using cumulative logit models, an extension of generalized linear models, as they did not present repeated measures over time. In both models, the effect of the treatment factor was assessed by likelihood ratio tests, comparing the null model with the model including the treatment effect. Comparisons between treatments were performed using the Bonferroni multiple comparisons test. All analyses were conducted using the R Software (R Core Team), with a significance level set at 5%. For the analysis of immunohistochemical variables, the reepithelialization variable, which is continuous quantitative, was evaluated using one-way analysis of variance (ANOVA). Tukey’s test was also applied to determine differences between factors.

## Results

### Macroscopic observation

The analysis of macroscopic variables (Table 1), including presence and degree of edema, presence and degree of erythema, presence and degree of pain/discomfort, vocalization upon manipulation, presence and degree of pruritus, presence and characterization of secretion, amount of secretion, and the progression of the healing process, showed no significant differences (*p* > 0.05) between the C group and the T1 and T2 groups.

Visual inspection of the wounds on the 10th day of treatment indicated a greater reduction in the diameter of the surgical wounds in the animals from the C group (Figs. 1, 2 and 3).

**Figure 1:**
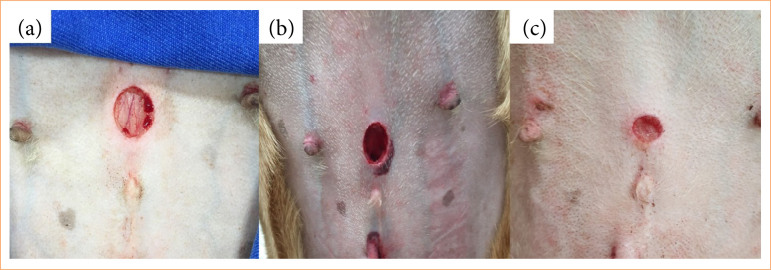
Photographic images of a surgical wound in the pre-umbilical region of the canine abdomen: skin incision of approximately 1 cm in length. (a) Wound at the time of creation, (b) on the first day of treatment, and (c) on the tenth day of treatment (group T1).

Source: Elaborated by the authors.

**Figure 2:**
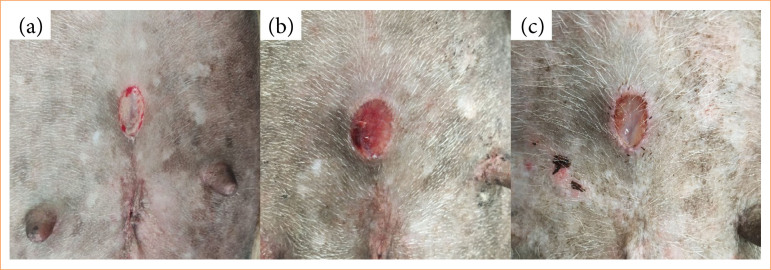
Photographic images of a surgical wound in the pre-umbilical region of the canine abdomen: skin incision of approximately 1 cm in length. (a) Wound at the time of creation, (b) on the first day of treatment, and (c) on the tenth day of treatment.

Source: Elaborated by the authors.

**Figure 3:**
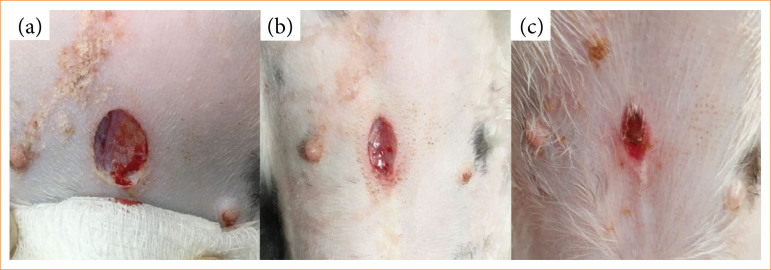
Photographic images of a surgical wound in the pre-umbilical region of the canine abdomen: skin incision of approximately 1 cm in length. (a) Wound at the time of creation, (b) on the first day of treatment, and (c) on the tenth day of treatment (group C).

Source: Elaborated by the authors.

### Microscopic observation

#### Histopathological evaluation

In the microscopic evaluation of histological slides stained with hematoxylin and eosin, features such as areas of necrosis, prominent inflammatory cell infiltration, loss of cellular architecture, vascular proliferation, moderate collagenization, fibroblast presence, and reepithelialization were observed in all groups. No statistically significant differences were found among the analyzed variables, except for collagenization, which was significantly higher in the C group compared to the T2 group. The T1 group did not differ significantly from either of the other two groups (Figs. 4, 5, 6, 7 and 8).

**Figure 4:**
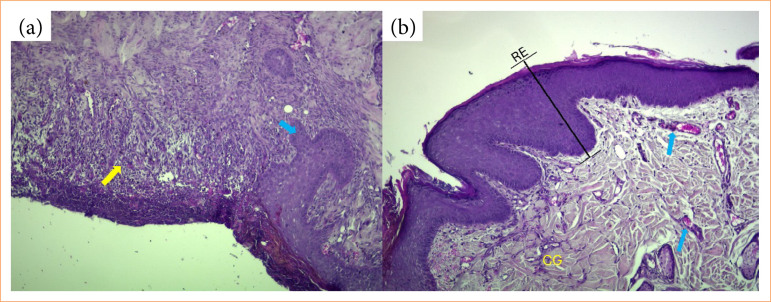
Photomicrographs of the epidermis and dermis of a dog treated with polyhexamethylene biguanide twice daily (T2 group). (a) Area of necrosis (yellow arrow) and viable tissue (blue arrow). (b) Area of reepithelialization, blood vessel presence (blue arrow), and collagenization (CG). Hematoxylin and eosin staining, 10× magnification.

Source: Elaborated by the authors.

**Figure 5:**
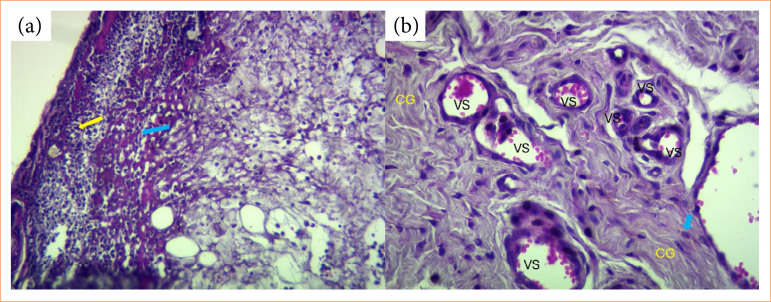
Photomicrographs of the epidermis and dermis of a dog treated with polyhexamethylene biguanide once daily (T1 group). (a) Area of necrosis with prominent inflammatory cell infiltration (yellow arrow) and loss of cellular architecture (blue arrow). (b) Blood vessel presence (VS), fibroblasts (blue arrow), and collagenization (CG). Hematoxylin and eosin staining, 20× magnification.

Source: Elaborated by the authors.

**Figure 6:**
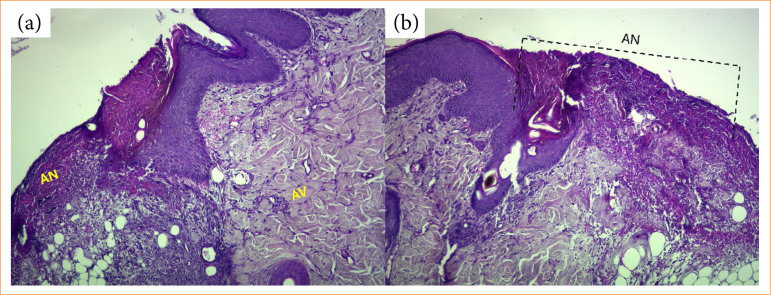
Photomicrographs of the epidermis and dermis of a dog treated with polyhexamethylene biguanide once daily (T1 group). (a) Area of necrosis (AN) and viable tissue with blood vessels and moderate collagenization (AV). (b) AN. Hematoxylin and eosin staining, 10× magnification.

Source: Elaborated by the authors.

**Figure 7:**
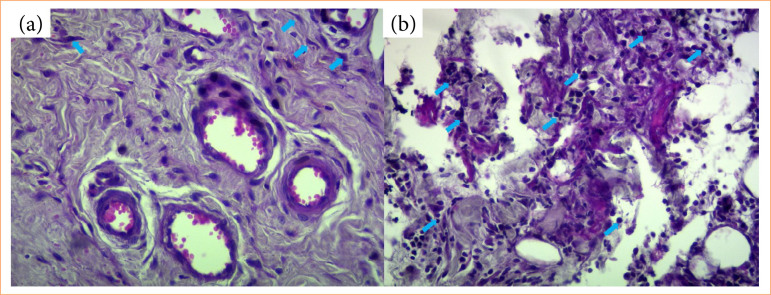
Photomicrograph of the epidermis and dermis of a dog treated with polyhexamethylene biguanide once daily (T1 group). (a) Fibroblast presence (blue arrow). (b) Inflammatory cell presence (blue arrow). Hematoxylin and eosin staining, 20× magnification.

Source: Elaborated by the authors.

**Figure 8:**
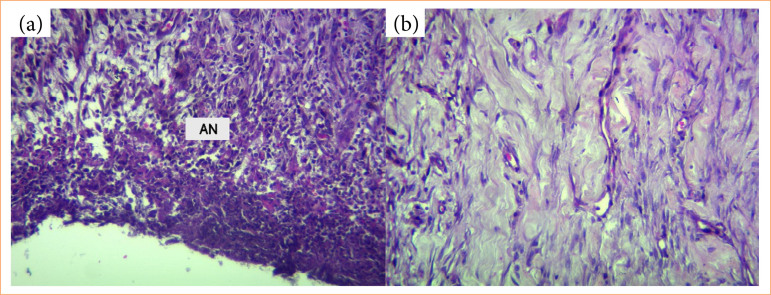
Photomicrograph of the epidermis and dermis of a dog treated with polyhexamethylene biguanide twice daily (T2 group), (a) showing an area of necrosis (AN). (b) Photomicrograph of the epidermis and dermis of a dog from the control group (C group), showing an area undergoing collagenization. Hematoxylin and eosin staining, 20× magnification.

Source: Elaborated by the authors.

#### Immunohistochemical evaluation

For the reepithelialization variable, different superscript letters indicate statistically significant differences between groups. In other words, on average, there was no significant difference between the C group and the T1 and T2 groups (Table 5). In the analysis of angiogenesis (blood vessel proliferation), the interpretation of letters in the tables and graphs follows the same approach. A significant effect was observed, with the number of vessels being higher in the C and T2 groups compared to the T1 group (Table 6).

**Table 5:** Descriptive analysis of the reepithelialization fraction variable and the effect of each treatment.

Variable	Treatment	n	Mean	SD	Min	Max	r
Reepithelialization	Control (C)	10	0.07ª	0.03	0.03	0.13	-
	1 PHMB (T1)	10	0.05ª	0.03	0.01	0.10	0.177
	2 PHMB (T2)	10	0.04ª	0.02	0.00	0.09	-

PHMB: polyhexamethylene biguanide; n: number of observations; SD: standard deviation; Min: minimum value; Max: maximum value; r: effect size. Source: Elaborated by the authors.

**Table 6:** Descriptive analysis of the number of blood vessels variable and the effect of each treatment.

Variable	Treatment	n	Median	IQR	Min	Max
Number of vessels	Control (C)	40	7b	2.97	3	15
	1 PHMB (T1)	40	5.5ª	2.22	2	13
	2 PHMB(T2)	50	7b	2.97	3	16

PHMB: polyhexamethylene biguanide; n: number of observations; IQR: interquartile range; Min: minimum value; Max: maximum value. Source: Elaborated by the authors.

## Discussion

The macroscopic and microscopic evaluations indicated that the use of dressings impregnated with PHMB gel did not yield superior results compared to the control group. Therefore, the hypothesis that PHMB would enhance wound healing was not confirmed.

In the macroscopic evaluations, no statistically significant differences were found among the variables in the three groups, suggesting that the healing process occurred similarly in all groups and that the use of the gel did not alter the visual appearance of clean surgical wounds. Positive results were reported by Sibbald et al.^10^ with PHMB use in stagnant human wounds.

In the histopathological evaluation of hematoxylin and eosin-stained slides, most analyzed variables showed no statistically significant differences between groups. Collagenization was the only variable showing a significant effect. The C group exhibited more intense collagenization compared to the T2 group, while the T1 group did not differ significantly from the other groups. This suggests that applying PHMB twice daily may have affected collagen formation, potentially causing a slight delay in wound healing, whereas applying PHMB once daily may have had an intermediate or comparable effect. According to Cialdai et al.^14^, fibroblast activity and all stages of collagenization are directly related to wound healing.

Maintaining moisture balance in the wound is essential, as desiccation can impair cell migration and proliferation, while excess moisture may lead to maceration of the wound margins and perilesional skin^10,15^. Notably, the two groups treated with gel-impregnated dressings maintained higher wound moisture, whereas the control group, treated with dry gauze, likely experienced lower moisture retention. Dry gauze may adhere to the wound bed and, upon removal, disrupt newly formed tissue and contribute to local desiccation, leading to a possible underestimation of the wound healing capacity in the control group. These factors may negatively affect the proliferative phase, which depends on fibroblast migration, collagen deposition, angiogenesis, and reepithelialization^16^.

According to Demidova-Rice^17^, blood vessel proliferation is a key feature of the healing process. Broughton et al.^18^ highlight the important role of inflammatory cells in tissue repair. Studies have reported that factors such as enhanced reepithelialization and reduced necrosis favor tissue healing^19,20^. In the present study, the use of PHMB gel-impregnated dressings did not significantly alter any of these variables compared to the control group. Furthermore, analysis of vessel numbers indicated that the effect of PHMB was similar to or less than that observed in the control group, suggesting that its efficacy in accelerating or improving the quality of healing was not demonstrated.

The use of PHMB gel-impregnated dressings did not significantly affect fibroblast proliferation, endothelial cell activity, or vascularization, indicating the absence of cellular toxicity and confirming its safety for use in dressings. This is consistent with *in-vitro* findings by Jin et al.^21^, who tested PHMB hydrogel dressings on non-infected rat wounds, and by Hirsch et al.^22^, which showed low cytotoxicity to keratinocytes and fibroblasts.

Babalska^23^ reported that PHMB is among the antiseptics recommended for human wound treatment, based on guidelines from the Polish Society for Wound Care and the German Consensus on Wound Antisepsis. In addition to its effectiveness in wounds with fluid stagnation, these findings suggest that PHMB-containing gel dressings may be more suitable for chronic or contaminated wounds. Extrapolation of these findings to surgical wounds should be made with caution, as they represent a distinct healing environment. The antimicrobial action of PHMB in surgical wounds was not evaluated in this study. Visual observations showed no signs of infection in any of the groups. However, no antimicrobial effect associated with PHMB use can be inferred, as microbial load was not directly assessed.

This study has important limitations that should be considered. Although macroscopic, histopathological, and immunohistochemical evaluations were performed, some unassessed variables could provide more precise information on the potential of PHMB gel-impregnated dressings in wound healing, including daily measurement of wound size, evaluation of different concentrations of the compound, and analysis of microbial load. In addition, the relatively small sample size and the duration of the evaluation period may have limited the ability to detect subtle differences between groups and to fully assess later stages of wound healing, such as tissue remodeling. Additionally, the use of dry gauze may have impaired the healing process in the control group, potentially leading to an underestimation of its healing capacity and influencing comparisons between experimental groups. The absence of these assessments limits a more detailed interpretation of treatment effects and comparison between groups over time. The lack of studies evaluating PHMB use in non-contaminated surgical wounds in dogs also limits comparison with the existing literature.

## Conclusion

Treatment of surgical wounds with PHMB gel-impregnated dressings did not result in superior outcomes compared to cleaning and dry gauze dressing. The findings suggest that PHMB does not impair key cellular aspects of wound healing and may represent a potential option for wound management in dogs.

## Data Availability

The data will be available upon request.
